# Cross-Sectional Evaluation of a Physiotherapist-Led Hip and Knee Osteoarthritis Program: Perceived Benefits and Factors that Influence Patient Access and Adherence

**DOI:** 10.1177/23743735261462001

**Published:** 2026-06-22

**Authors:** Nikole Watson, Hayley Legg, Alison Irvine, Rebecca Sawatsky, Brenna Bath

**Affiliations:** 1 School of Rehabilitation Science, 7235 University of Saskatchewan, Saskatoon, Canada; 2 Craven SPORT services, Saskatoon, SK, Canada; 3 School of Education and Applied Sciences, 2376 University of Gloucestershire, UK

**Keywords:** osteoarthritis, patient experience, physiotherapy, group exercise, adherence, health services accessibility

## Abstract

Osteoarthritis (OA) affects millions of Canadians with substantial healthcare costs, yet education and exercise can improve outcomes. This study explored the perceived benefits, facilitators, and barriers influencing perceived access and adherence to an 8-week physiotherapist-supervised exercise and education program in a private clinic. Adults with hip and/or knee OA enrolled in the program were invited to complete a cross-sectional online survey. Descriptive statistics and qualitative content analyses were used to describe participant demographics, clinical features, and perceptions. Of 102 participants, 61 completed the survey (60% response rate). Perceived benefits identified by participants included physical improvements, socialization, increased confidence, expert support, and improved self-efficacy. Perceived facilitators supporting adherence were physical ability, financial support, transportation, and provider expertise. While most participants reported no major barriers, some noted financial constraints, physical limitations, inconvenient class times, and work demands. A group-based hip and knee OA program delivered by physiotherapists can provide substantial perceived physical, psychological, and social benefits, though self-reported adherence may be influenced by financial, physical, and social factors.

## Plain Language Summary

**Why did we do this study?** Osteoarthritis (OA) can cause pain and make daily activities difficult. While supervised exercise and education programs can help, we wanted to understand the patient experience. We wanted to understand directly from patients with hip and knee arthritis what they felt were the benefits of a physiotherapist-led group program, as well as what made it easier to attend and what challenges got in the way.

**How did we do this study?** We invited adults with hip or knee OA who were enrolled in an 8-week group program at a private-practice clinic to complete an online survey about their experiences. We asked about the positive changes they noticed and what helped or hindered their ability to stick with the program.

**What did we find?** Participants reported benefits beyond just physical improvements. While they felt stronger and moved more easily, they also highlighted the value of social connection with others in the group. Many felt more confident and less afraid of their pain. The expert guidance from physiotherapists was crucial for helping them feel safe and motivated. Facilitators included physical ability, financial support from health insurance, and reliable transportation. The most common challenges were the program’s cost, especially after insurance benefits ran out. Other barriers included pain and class times conflicting with work schedules.

**What does this mean?** Group exercise programs led by physiotherapists can provide whole-person care, helping patients physically, socially, and emotionally. For these programs to help more people, our findings show we must address practical challenges like program cost and scheduling flexibility.

## Introduction

Osteoarthritis (OA) affects over 4.6 million Canadians, with healthcare costs exceeding $1.4 billion annually.^1,2^ Saskatchewan residents face some of the longest wait times in Canada for total joint arthroplasty (TJA),^3^ leaving many without timely surgical or non-surgical care. Conservative approaches including education and exercise are effective for moderate to severe OA, reducing pain and improving function and quality of life.^4^ Without conservative care, individuals may adopt compensatory movement patterns, worsening joint degeneration and mobility loss.^5^ By helping individuals with OA maintain physical abilities, improve movement strategies, and manage pain, exercise can lead to better daily function and post-surgical outcomes.^6^ When delivered through physiotherapist-led programs that combine exercise and education, this approach has proven effective for those with mild to severe hip or knee OA.^7^ While the clinical efficacy of these conservative approaches is well-established, there is a critical need to understand the patient-reported factors that translate clinical guidelines into sustainable, everyday management.

This study aims to identify the perceived benefits of a physiotherapist-led exercise and education program designed for individuals with hip or knee OA, and how participant demographics and perceived facilitators and barriers may be associated with perceived program access and self-reported adherence. The objectives of this study were to: 1) describe the sociodemographic and clinical participant features, and 2) identify and categorize perceived benefits of program participation and the perceived facilitators and barriers which impact adherence. By utilizing a patient-oriented approach to identify perceived benefits, facilitators, and barriers, this research advances patient-centered care by aligning research priorities with lived experiences, providing insight into the drivers of successful long-term self-reported adherence to conservative care. Understanding these factors could inform future program development and delivery, helping to guide the implementation of similar programs by identifying strategies to enhance perceived accessibility and engagement across diverse populations.

## Methods

### Study Design

This study adheres to the STROBE (STrengthening the Reporting of OBservational studies in Epidemiology) cross-sectional reporting guidelines^8^ and evaluated participant experiences in an OA exercise and education program. The outcomes of interest were participant-perceived benefit from the program and perceived facilitators and barriers to program access and self-reported adherence. To ensure these outcomes aligned with the priorities of individuals with OA, this study utilized a patient-oriented approach, which integrates patients as collaborators with clinicians and researchers to produce evidence that directly improves care.^9^ Three patient partners (i.e. program participants with lived experience of hip or knee OA) contributed to survey development, interpretation of findings, and manuscript preparation. In this study, ‘Access’ was defined as the participant’s perceived ability to enter and participate in the program, whereas ‘Adherence’ was qualitatively measured and defined by the self-reported participation duration and subjective identification of factors that influenced continued engagement, rather than objective attendance logs.

### Program Participants and Setting

Study participants were individuals living with OA who were enrolled in the OA program. The OA program is a physiotherapist-led initiative tailored for individuals with hip or knee OA and delivered in a private-practice setting. Using an evidence-based approach, physical therapists deliver twice-weekly group classes over eight weeks to support OA management.^10^ The program emphasizes education, neuromuscular and strength development, functional movement training, and ongoing progress monitoring to help individuals reduce symptoms and restore function.

### Eligibility Criteria

Inclusion criteria consisted of current OA program participants aged 18 or older with self-reported hip or knee OA (i.e. diagnosis was self-reported and not verified through imaging or clinical assessment).

### Data Collection

Participants who provided online informed consent were invited to complete an anonymous online survey about their experience in the program. A survey link was generated through REDCap, a secure, online, web-based platform^11,12^ hosted at the University of Saskatchewan and distributed via email to individuals with hip and/or knee OA who were current program participants. Invitations were sent twice between October and December 2023. A range of self-reported variables were collected via close-ended (quantitative) and open-ended (qualitative) survey questions to explore their potential influence on perceived outcomes and adherence. These include sociodemographic variables (age, gender, education, income, residence, employment status, health insurance status) and other variables (self-reported height and weight, number and type of joints affected, prior treatments excluding total joint arthroplasty [TJA], history of TJA, plans for TJA, and other health conditions. A description of these variables can be found in Table 1.

**Table 1.** Description of Study Variables

Variable	Units/Variables	Description (if applicable)
**Demographic and Health Information**		
Age	Years	N/A
Education	Completed at least some post-secondary education or less	Self-reported highest level of education (eg. trade school, some college courses, a college diploma, some university courses, high school, or no completion of grade twelve)
Annual income	Canadian dollars	Household annual income
Residence	Rural	The second digit is 0 in postal code^13^
Employment status	Working	Paid work full time, paid work part time, or self-employed
Additional health insurance	N/A	Benefits through employer or personal health insurance
Other bone/joint problems	N/A	Tendon tears or tendinitis, surrounding joints affected from other joint replacements, osteoporosis, previous dislocations or fracture, kyphosis, scoliosis, or low back issues
Other health issues	N/A	Rheumatoid arthritis, hypothyroidism, high cholesterol, recurring cellulitis, sleep issues, hyperthyroidism, multiple myeloma, chronic kidney disease, autoimmune disorders, benign paroxysmal positional vertigo, Fuchs corneal dystrophy, migraines, burnout, diabetes, hereditary angioedema
None	N/A	No other health conditions reported
BMI	Kg/m^2^	Body mass index categorized by the World Health Organization^14^
**OA Characteristics and Interventions**		
Symptom duration	Months	Duration of months experiencing hip or knee symptoms related to OA
Program attendance duration	Months	Duration of months participating in the OA program
**Prior Treatment for OA (excluding TJA)**		
Over-the-counter medication	N/A	Non-prescription medication that can be used to manage OA symptoms
Prescribed medications	N/A	Prescription medication that can be used to manage OA symptoms
Injection	N/A	Injectable medication that can be used to manage OA symptoms (eg. cortisone)
Other	N/A	Acupuncture, deep water aquacise, neuro-stimulation implant, bracing
None	N/A	No other prior treatments reported
**Benefits, Facilitators, and Barriers**		
Benefits	N/A	Perceived benefits of the OA program
Other benefits	N/A	Reduced feelings of loneliness, ability to defer joint replacements, and receiving expert supervision from physiotherapists.
Facilitators	N/A	Perceived factors that positively influenced program participation
Other facilitators	N/A	Program quality and familiarity with gym settings from prior experiences.
Barriers	N/A	Perceived factors that negatively influenced program participation
Other barriers	N/A	Low motivation, vacation/holidays, and illness.
None	N/A	No other benefits, facilitators, or barriers to report

OA; osteoarthritis, TJA; total joint arthroplasty.

### Data Analysis

Descriptive statistics were used to summarize participant demographics, clinical characteristics, and program-related variables. Frequencies and percentages were reported for categorical variables. Quantitative analysis was conducted by a single reviewer using Microsoft Excel to compute summary statistics. No inferential or hypothesis-driven statistical tests were performed due to the descriptive nature of the study. Responses to open-ended questions were analysed using NVivo^15^ qualitative analysis software. Two researchers independently conducted inductive thematic coding to identify recurring patterns in the data. To ensure rigor, researchers met to compare codes and resolved any disagreements through consensus-based discussion. This collaboration aimed to minimize individual interpretive bias and ensure the final themes accurately represented the participants lived experience.

### Ethical Considerations

The study was approved by the University of Saskatchewan Behavioural Research Ethics Board (ID 4102), Saskatchewan, Canada, and was conducted in accordance with relevant national ethical guidelines. All participants provided informed consent before completing the survey.

## Results

A total of 102 individuals enrolled in the OA program between October and December 2023 were invited to participate in the survey. Of these, 61 participants (60% response rate) completed the survey and met all inclusion criteria. The remaining 41 individuals did not complete the survey and reasons for non-participation or demographic information were not collected. This lack of data on non-responders limits our ability to assess the direction or magnitude of the non-responder bias and makes it impossible to determine if the 61 participants who did respond are truly representative of the entire program enrollment. Additionally, there were incomplete survey responses among some participants resulting in missing data; the number of respondents for each variable is indicated in Table 2 and Table 3.

**Table 2.** Demographic, Health Characteristics, Osteoarthritis Status, and Prior Treatments Among Participants

Variable	Participants, no. (%)	Mean ± SD	*n* *
**Demographic and health information**			
Age			
<55 years	6 (9.8)	46.8 ± 3.3	61
≥55 years	55 (90.2)	66.0 ± 7.5	61
Age range (min – max)	​	42 – 86	​
Education	25 (41.0)	​	61
Annual income			
< $15,000	0 (0.0)	​	54
$15,000–59,999	15 (27.8)		54
$60,000–99,999	18 (33.3)		54
≥$100,000	21 (38.9)		54
Rural residence	4 (6.7)		60
Employment status: working	20 (32.8)		61
Additional health insurance	50 (82.0)		61
**Other health problems:**			
Respiratory, circulatory, or gastrointestinal issues	17 (27.9)	​	61
Mental health	8 (13.1)		61
Other bone/joint problems	12 (19.7)		61
Other health issues	16 (26.2)		61
None	13 (21.3)		61
BMI			
<18.5 (Underweight)	1 (1.7)	​	58
18.5–24.9 (Healthy weight)	16 (27.6)		58
25.0–29.9 (Overweight)	21 (36.2)		58
≥30.0 (Obese)	20 (34.5)		58
BMI range (min – max)	​	16.5–52.7	​
**OA Characteristics and Interventions**			
Symptom duration in months	​	109 ± 107.8	61
**Number of body regions with OA:**			
1	16 (26.7)	​	60
2	11 (18.3)		60
3	15 (25.0)		60
4	11 (18.3)		60
5 or more	7 (11.7)		60
**Most common OA locations:**			
Bilateral knees	28 (46.7)	​	60
Bilateral hips	15 (25.0)		60
A knee and a hip	15 (25.0)		60
Bilateral knees and bilateral hips	6 (10.0)		60
**Joint replacement:**			
Received TJA	13 (21.3)	​	61
Plan for future TJA	40 (65.6)	​	61
Program attendance duration in months	​	9.45 ± 13.8	61
**Prior Treatment for OA (excluding TJA)**			
Physiotherapy	52 (85.0)	​	61
Massage	32 (52.0)		61
Chiropractic	22 (36.0)		61
Over-the-counter medications	44 (72.0)		61
Prescribed medications	20 (33.0)		61
Injection	28 (46.0)		61
Other	5 (8.0)		61
None	4 (7.0)		61

OA, osteoarthritis; BMI, body mass index; TJA, total joint arthroplasty.

*Each variable has a different *n* as some participants chose not to answer certain questions.

**Table 3.** Perceived Benefits, Facilitators, and Barriers to Self-Reported Program Adherence

Variable	Participants, no. (%)
**Benefits (*n*=60)**	
Physical improvements	55 (91.7)
Socialization	44 (73.3)
Increased confidence with managing OA	42 (70.0)
Pain management	36 (60.0)
Increased understanding of OA	34 (56.7)
Feelings of hope for the future	28 (46.7)
Increased functional independence	27 (45.0)
Improvements of other health conditions	15 (25.0)
Other	3 (5.0)
None	1 (1.7)
**Facilitators (*n*=61)**	
Physical ability	40 (65.6)
Health insurance or financial support	30 (49.2)
Reliable transportation	28 (45.9)
Family/home support	25 (41.0)
Community/group setting provided	16 (26.2)
Other	2 (3.3)
None	6 (9.8)
**Barriers (*n*=61)**	
Financial	13 (21.3)
Pain or physical inability	10 (16.4)
Program times offered not suitable	4 (6.6)
Other	4 (6.6)
No available space in classes	3 (4.9)
Caregiver responsibilities	2 (3.3)
Transportation	1 (1.6)
None	34 (55.7)

Participant demographic, health, and osteoarthritis-related characteristics are reported in Table 2. While most variables had complete data, missing responses were noted for residence (n = 1), body mass index (n = 3), and annual income (n = 7). Non-exclusive response options were used for close-ended questions to capture participants’ perceptions of program benefits, facilitators, and barriers to self-reported adherence. These responses reflect multiple, co-occurring experiences as participants could select all applicable item options. Quantitative frequencies are presented in Table 3, while qualitative themes and illustrative participant quotes in Table 4 contextualize the findings. Additional representative quotations are included in Appendix A.

**Table 4.** Participant-Reported Benefits, Facilitators, and Barriers by Theme (Qualitative Quotes)

Theme	Participant quotes
**Benefits**	
Physical improvements	“Muscle strength and mobility has improved. Greater flexibility in daily activities and increased endurance. Huge difference in day-to-day activities.”
	“This program has given me my mobility back. When I began last January, I could hardly walk a block.”
Symptom management	“I was on a waiting list for a second hip and knee replacement. X-rays showed the same bone on bone conditions. I deferred and was taken off the waiting list 15 months later because the pain level in the second joint is now ‘1′ with no pills.”
	“I believe my mobility and pain management would be negatively affected without the OA program, and I would be in worse physical and mental shape because of it.”
Improved OA management	“I have learned what Osteoarthritis is and how strengthening the muscles and tissue around my knees and hips and back can prevent the joint pain.”
	“I am more confident in my mobility. I am no longer afraid of my pain and no longer allowing it to keep me back from certain activities.”
Expert guided support	“Having a physiotherapist set up group program, see you twice weekly and modify exercises was crucial. Building that relationship allowed me to push further than I thought possible.”
Socialization and support	“I loved the group setting and found it gave me hope as I saw people worse off and better off than me. I would look at them and think, ‘they know how hard this is.’ It gave me comfort.”
Improved self-efficacy	“Helped build confidence and sense of self as healthy, capable, and strong.”
**Facilitators**	
Health-care provider support	“The instructors are a big part of the reason I’ve stayed with the program for nearly 2.5 years. They are helpful, encouraging, patient, and have never made me feel stupid for struggling or for being overweight.”
Physical ability	“Results from the first two months of the program encouraged further attendance.”
	“I see progress in mobility and pain. Also see improvements in performing the exercise as well.”
Socialization	“I enjoyed the group setting with others who are experiencing similar issues. We helped and encouraged each other!”
Familial support	“My husband is supportive and encourages me to attend, and my children, who live in Saskatoon, often offer me the option of staying there when I don’t feel like travelling on two separate days.”
Financial support	“My private health insurance pays the first $350 per year. Just a portion but it helps.”
Transportation	“Easy to get to with vehicle.”
Barriers	
Financial concerns	“Retired on a fixed income.”
	“My annual physio benefits are $300. That ran out in June. I’ve been paying the additional program costs.”
Physical inability	“There have been sessions missed due to pain.”

OA, osteoarthrosis; HCP, health care provider.

Most participants (77%) were women aged over 55, with 71% classified as overweight or obese. Approximately three-quarters reported a household income above $60,000 and had additional health insurance. Bilateral knee OA (n = 28, 47%) was the most frequently reported condition, and comorbidities were common. Many participants had previously received physiotherapy or over-the-counter medications as non-surgical treatments. Summary statistics and categorical distributions are presented in Table 2.

### Thematic Definitions

Analysis of the open-ended survey responses revealed several themes regarding participant experiences. To ensure a shared understanding of these findings, the themes are defined based on the core experiences reported by participants.

Perceived benefits were defined by five key themes: Physical improvements (reported gains in mobility, strength, or function); Symptom management (reductions in pain or swelling); Improved OA management (the acquisition of disease-control strategies); Expert-guided support (the value of professional physiotherapist oversight regarding education and exercise); Socialization and support (the motivational impact of peer engagement in a group setting); and Improved self-efficacy (the increase in confidence toward independent condition management).

Perceived facilitators to adherence were defined by four key themes: Support systems (the encouraging influence of healthcare providers, family members, or peers); Physical ability (a baseline level of health that permitted successful program engagement); Socialization (the desire for community or group interaction and shared experiences); and Practical determinants (the availability of enabling resources such as reliable transportation and financial means).

Perceived barriers to adherence were characterized by two key themes: Financial concerns (the perceived economic burden of program costs or a lack of adequate insurance coverage); and Physical inability (health-related limitations, such as acute symptom flare-ups or co-morbidities).

### Perceived Benefits, Facilitators, and Barriers

Participants identified a range of perceived benefits from the program, with the most frequently reported being physical improvements (91.7%), social connection (73.3%), and increased confidence in managing OA (70.0%) (Table 3). As one participant stated, *“Muscle strength and mobility has improved. Huge difference in day-to-day activities.”* Another shared, *“This program has given me my mobility back.”* Pain management (60.0%) and improved understanding of OA (56.7%) were also common benefits. One participant noted, *“The program has been beneficial in reducing arthritis pain and inflammation in my knees,”* while another said, *“I am no longer afraid of my pain and no longer allowing it to keep me back from certain activities.”* Participants frequently highlighted the importance of expert guidance from physiotherapists. One noted, *“Building that relationship allowed me to push further than I thought possible,”* and another described the *“excellent, tailored instruction for my specific mobility and strength issues.”*

Beyond a benefit, social interaction emerged as a key perceived facilitator of self-reported adherence, with 26.2% of participants citing the group setting as supportive (Table 3). Participants valued the peer support and shared experience: *“I enjoyed the group setting with others who are experiencing similar issues.”* and *“I loved the group setting and found it gave me hope.”* Other commonly reported perceived facilitators included physical ability (65.6%), financial support (49.2%), and access to transportation (45.9%). Physical progress reinforced motivation for many, as one participant explained, *“Results from the first two months of the program encouraged further attendance.”* Support from healthcare providers was also critical: *“Having a physiotherapist ensure I was performing the exercises correctly gave me the confidence to continue.”*

While 56% of participants reported no barriers to participation, others identified several perceived challenges, including financial constraints (21.3%), physical limitations (16.4%), inconvenient class times (6.6%), and competing responsibilities (Table 3). Financial concerns were common, with one participant stating, *“I’ve maxed out my private insurance coverage,”* and another noted, *“My annual physio benefits is $300. That ran out in June. I’ve been paying the additional program costs.”* Physical symptoms also interfered with attendance: *“There have been sessions missed due to pain.”* Others noted life demands: *“Job demands caused me to miss some classes.”* Among those who did report difficulties, financial constraints and physical symptoms were the most frequently cited factors perceived to influence self-reported attendance.

## Discussion

This cross-sectional survey of 61 participants of a physiotherapist-led group-based education and exercise osteoarthritis program identified participant sociodemographic and clinical characteristics, as well as perceived benefits, facilitators, and barriers to perceived access and self-reported program adherence. Participants were predominantly women (77%) and older adults (mean age: 66.0 ± 7.5 years), with most having a BMI in the overweight or obese range. The majority reported annual incomes above $60,000, had supplementary health insurance, and lived in urban areas; one-third were employed. Bilateral knee OA was the most reported condition, and many participants also reported experiencing comorbidities. Participants reported a range of benefits from the program, most notably physical improvements, socialization, and increased confidence in managing osteoarthritis. Key perceived facilitators included physical ability, financial support or insurance, and reliable transportation. Perceived barriers were less frequently reported, but included financial challenges, pain or physical limitations, and inconvenient program times.

Our participant’s demographics, predominantly female and averaging 66 years of age, closely resemble those in the GLA:D® Canada program.^16^ Our cohort of primarily urban, insured adults also shares key characteristics with participants in the Better Knee, Better Me™ trial, which involved adults with knee OA and elevated BMI.^17^ In contrast, the ESCAPE-pain study enrolled a more socioeconomically diverse population from public healthcare settings,^18^ whereas our sample was drawn from a fee-based clinic. This distinction is important, as our largely urban sample may not reflect the significant access challenges, such as substantially longer travel times, often faced by rural Canadians with OA.^19^ The limited rural representation in our cohort, where 56% of respondents reported no participation barriers, suggests the program’s urban location, participant’s higher income, and insurance coverage helped mitigate common perceived access challenges. Although clinically similar to the broader OA population, the sample’s socioeconomic advantages limit the findings’ generalizability to those facing greater financial or geographic barriers.

The perceived benefits reported by participants emphasize the holistic impact of engaging in a structured education and exercise program for osteoarthritis, with reported benefits encompassing physical, social, and emotional domains. Perceived physical improvements include increased strength and mobility, improved function with daily activities, and reduced pain. These outcomes are relevant considering individuals with OA commonly experience greater pain, functional limitations, and chronic health conditions compared to the general population, with many being overweight, obese, or physically inactive.^20^ Many individuals with OA enter a cycle where pain leads to inactivity, resulting in further declines in mobility and function, which in turn exacerbates symptoms and sustains inactivity.^21,22^ Structured exercise programs are a key component of non-surgical OA management,^22^ offering a pathway to disrupt this cycle and support improved physical function.

In addition to self-reported physical improvements, participants described psychosocial benefits. Similar studies report that individuals in group-based exercise therapy value the support and connection, which helps reduce isolation and foster hope.^23^ Improved understanding of OA and physical progress can contribute to greater self-efficacy,^23^ highlighting the need to address psychosocial factors alongside physical outcomes. Our findings support this evidence, with participants highlighting a range of psychosocial benefits that influenced their engagement in the program.

Effective OA management requires collaborative patient–healthcare provider relationships, adequate disease education, and active patient engagement. Psychosocial factors such as pain catastrophizing, health literacy, and self-efficacy significantly impact patient compliance and adherence to interventions.^24^ However, fewer than 25% of individuals receive sufficient education on OA diagnosis, management, and disease progression,^24-27^ limiting their capacity for self-management. Disease-specific education and consistent feedback from professionals has shown to manage expectations, identify progression risk factors, and support adherence.^6^ Our findings support this evidence by demonstrating the value of patient-centred care within a physiotherapist-led OA program, reinforcing the importance of personalized, clinician-led care for improving adherence and outcomes. The current study identified expert supervision and guidance as key benefits, with participants expressing greater confidence in challenging their physical limits after receiving education on the purpose and outcomes of exercise. This highlights the need for healthcare providers to deliver tailored, disease-specific education that reflects patients’ health literacy and evolving needs.^24,28^

Several common perceived facilitators and barriers emerged from participant responses. Financial support was viewed as facilitating self-reported program adherence, while financial concerns were a perceived barrier. Although many participants had additional health insurance, some noted it only covered one or two sessions, requiring them to pay for continued care. These findings align with previous research identifying private health insurance as an enabler of access to non-surgical treatments such as physical therapy, and even individuals with insurance face significant financial barriers due to limited coverage and high out-of-pocket costs.^29^ Furthermore, financial strain can deter ongoing participation and raises concerns about the sustainability of self-funded treatment.^29^ These shared perspectives reinforce the need to address financial accessibility in program design to support long-term engagement with non-surgical OA interventions.

Engagement in OA exercise programs is influenced by personal and social factors. Prior research has identified personal beliefs about exercise outcomes and capabilities as common barriers, while structured environments that offer reinforcement and support act as key facilitators.^30^ Our findings align with this, as participants frequently reported physical improvements that motivated continued attendance, highlighting the value of structured programming. Structured interventions can improve symptom control, self-efficacy, and reduce fear of movement in individuals with musculoskeletal (MSK) conditions.^31^ Similarly, our participants described gaining confidence in their physical abilities and a greater sense of control over their OA symptoms. Program engagement is critical to success, and previous studies show that lower adherence to physical therapist-led OA interventions is associated with poorer outcomes,^23^ while supervised, structured programs that incorporate monitoring, feedback, and education have been shown to improve adherence.^32,33^ Participants in our study echoed these findings, highlighting expert support and feedback as central to their experience and naming expert guidance as the most reported qualitative benefit. While cost, location, and wait times have been cited as barriers to accessing physiotherapy in other MSK populations,^31^ our participants primarily reported financial strain, with fewer geographic concerns, likely due to the program’s urban setting. Notably, 56 % of participants reported no perceived barriers, highlighting the role of strong client–therapist relationships and supportive environments in supporting perceived engagement with non-surgical interventions.

Future program planning should consider delivery models that address cost and perceived service accessibility. Group-based programs offer a cost-effective alternative to private individual physiotherapy, with evidence showing comparable outcomes in pain reduction, functional improvement, and patient satisfaction among individuals with musculoskeletal disorders.^34^ In addition to potential cost savings and reduced access to care wait times, group formats have been shown to promote socialization, which can enhance motivation and adherence.^35,36^ Our findings align with this evidence, as participants frequently cited the affordability, peer support, and motivating group environment as key perceived facilitators of engagement in the program.

While this study highlights the benefits for those enrolled, the identified barriers provide insight into populations likely excluded from this model. The theme of financial concerns suggests that individuals without extended health insurance or sufficient disposable income may be unable to access private-practice, physiotherapist-led programs. Consequently, this model may primarily serve higher-resource populations, potentially widening the gap for those of lower socioeconomic status who rely on publicly funded care.^3,31^ Furthermore, the barrier of physical inability, characterized by acute flare-ups and comorbidities, indicates that patients with the highest disease severity or complex medical needs may be excluded from a standardized group exercise format.^23^ Since the program was delivered in a private-practice setting, geographic location may have acted as a perceived barrier. As noted in the facilitators, participants highlighted the importance of reliable transportation, suggesting those in rural areas may perceive significant barriers to access due to a lack of accessible transit options.^19,31^ By acknowledging these perceived barriers, it becomes clear that while the program is effective for those who can attend, further research is needed to adapt this model for more marginalized or medically complex populations.^16,29^

## Strengths and Limitations

Several measures were taken to minimize potential bias in the study and enhance the reliability of findings. To reduce selection bias, all eligible participants were invited to complete the survey, which was administered anonymously and independently to limit social desirability bias. Data were collected during active program participation to reduce recall bias, and standardized, structured questions were used to improve consistency. Qualitative responses were independently coded by two researchers using NVivo to enhance coding reliability and limit interpretive bias. The survey achieved a 60% response rate—exceeding the 52.7% average found across 490 published cross-sectional studies using individual-level questionnaires,^37^ aligning with the ≈60% mean for mail surveys in medical journals,^38^ and meeting the ≥60% threshold recommended for program evaluation research.^39^ This strong response rate reduces the risk of non-response bias while enhancing the precision and generalizability of the descriptive findings within the sampled population.^40^

Several limitations should be interpreted with caution given the descriptive nature of the study. The cross-sectional design limits the ability to draw causal inferences, as data were collected at a single time point; consequently, the predominance of reported perceived benefits over barriers is likely influenced by positivity and self-selection biases. Due to the study design, participants who found the program ineffective or had lower attendance due to barriers were less likely to be captured in the sample. This implies that the high satisfaction and adherence levels reported here likely reflect a ‘best-case’ scenario among successful participants rather than the experience of every individual who was enrolled. These biases suggest that while the program can produce substantial benefits, these results should be interpreted as the perceived potential of the program under optimal conditions (e.g., higher income, urban access, and high motivation). Readers should exercise caution when applying these findings to broader public health settings where the ‘non-responder’ profile, those with lower income or higher disease severity, is more prevalent.^29^

Additionally, because demographic and clinical data were not available for the 40% of invited individuals who did not complete the survey, the extent of non-responder bias cannot be fully determined. Although our response rate meets established thresholds for program evaluation research,^39^self-reported data remain subject to recall and social desirability biases, which could further overestimate favorable outcomes. Furthermore, the reliance on self-reported OA diagnosis introduces the risk of misclassification, as participants’ symptoms were not verified through clinical assessment or imaging. This may affect the generalizability of the results, as the cohort could potentially include individuals with non-OA joint pain or inflammatory conditions whose experiences with exercise may differ from those with confirmed osteoarthritis. We also did not correlate survey responses with objective attendance records or completion rates; consequently, ‘adherence’ reflects the participants’ perspective of their consistency and longevity in the program rather than a verified clinical metric. Given the private urban setting and the high proportion of participants with additional health insurance, findings from the small sample size may not generalize to rural populations or individuals without insurance.^31^ Rural residents often face distinct barriers, such a longer travel times,^19^ which were not fully captured in this study. Some variables, such as income, residence, and BMI, had missing data, which may limit the representativeness of the reported participant characteristics. Further research using longitudinal designs and comparative analyses is needed to clarify the extent to which these findings represent the broader population.

## Conclusions

Multiple factors may influence perceived access to osteoarthritis exercise programs, including demographic, physical, and psychosocial determinants. Participants in the OA program reported perceived benefits that addressed physical, social, and emotional needs, reinforcing the importance of a biopsychosocial approach. Program accessibility may be enhanced by financial assistance and group-based delivery models led by qualified professionals. Self-reported adherence was perceived to be promoted by physiotherapists who foster self-efficacy through education and feedback. This study supports existing evidence that expert guidance and social support are key to participant engagement and improving outcomes in OA programs. However, given the descriptive design and sociodemographic profile of participants, findings should be interpreted cautiously and may not generalize to underserved populations. Future studies should explore long-term outcomes and applicability across more diverse settings to inform broader implementation.

## Data Availability

The data that support the findings of this study are available from the corresponding author, Nikole Watson, upon reasonable request.*

## Appendix

**Appendix A.** Additional Participant-Reported Benefits, Facilitators, and Barriers by Theme

Theme	Participant quotes
Benefits	
Physical improvements	“I feel that my arthritis has progressed, and I still have daily knee pain, but I am also stronger, and I move more functionally so the arthritis is manageable.”
	“I am not able to have joint replacement surgery, so I need this program to help with joint movement.”
Symptom management	“The program has been beneficial in reducing arthritis pain and inflammation in my knees.”
	“Being taught ways to move that can reduce the pain.”
Improved OA management	“I am able to walk longer without pain medication.”
Expert guided support	“High ratio of physical therapists to supervise/coach. One to one assistance when needed. Very knowledgeable and skilled staff who listen and modify program as needed.”
	“The expert skill level coupled with the amazing optimism and support of the physiotherapist, and his helper has been key in building my confidence and ensuring success.”
	“Exercising safely with educated, respectful, and polite instructors is of extreme benefit. Without direct instruction and knowledge, regarding repetition and weight ratios, there would be limited chances of improvement and risk of injury.”
	“Small group size and good attention from instructors.”
	“I really appreciate the individual attention and feel I am getting the best possible results for my time and money.”
	“The instruction and guidance for each part of the program were excellent, tailored to my specific mobility and strength issues. I was shown how to adapt exercises, correct technique, and how to use the mirror to check progress.”
	“The enthusiasm, skill and confidence of the physios leading the groups is remarkable. They really are encouraging, and they love their work, very dedicated.”
Socialization and support	“Social aspect of group important.”
Improved self-efficacy	“Able to transfer exercises learned to a home practise.”
	“Taking responsibility for improving life, mobility and independence!”
Health-care provider support	“The compassionate approach and detailed explanations made it very easy to participate.”
	“Physio led specifically for my problem joints. The program is varied and never boring. Having a second instructor is very helpful.”
	“The confidence that a physiotherapist is making sure I’m doing the exercises correctly.”
Physical ability	“I see progress in mobility and pain. Also see improvements in performing the exercise as well.”
	“I’m mobile.”
	“I need to get more strength.”
Socialization	“There is a good vibe in our group as most of us have been together for 2+ years.”
	“Encouraging welcoming programs. Similar age group and everyone has similar issues.”
	“Nice group setting.”
Familial support	“My children are all health care providers of some kind. And strongly suggested I go.”
Financial support	“I have insurance.”
Transportation	“I have a car.”
Barriers	
Financial concerns	“Not covered by Blue Cross. Very expensive.”
	“I’ve maxed out my private insurance coverage.”
	“Not a financial drain, however, to continue with other phases it will get more expensive.”
Physical inability	“Neck pain/migraine.”
Class times	“I would prefer morning classes as opposed to afternoon classes.”

## References

[1] Vos T Flaxman AD Naghavi M , et al. Years lived with disability (YLDs) for 1160 sequelae of 289 diseases and injuries 1990–2010: a systematic analysis for the Global Burden of Disease Study 2010. The Lancet. 2012;380(9859):2163-2196. doi:10.1016/S0140-6736(12)61729-2. PMC6350784 23245607

[2] Bone and Joint Canada » Osteoarthritis. Accessed June 25, 2024. https://boneandjointcanada.com/osteoarthritis/

[3] Wait times for priority procedures in Canada, 2022, CIHI. Accessed June 25, 2024. https://www.cihi.ca/en/wait-times-for-priority-procedures-in-canada-2022 10.12927/hcq.2024.27437 39492731

[4] Kolasinski SL Neogi T Hochberg MC , et al. 2019 American College of Rheumatology/Arthritis Foundation Guideline for the Management of Osteoarthritis of the Hand, Hip, and Knee. Arthritis Rheumatol. 2020;72(2):220-233. doi:10.1002/art.41142. 31908163 PMC10518852

[5] Shakoor N Hurwitz DE Block JA Shott S Case JP . Asymmetric knee loading in advanced unilateral hip osteoarthritis. Arthritis Rheum. 2003;48(6):1556-1561. doi:10.1002/art.11034. 12794823

[6] Thorstensson CA Garellick G Rystedt H Dahlberg LE . Better Management of Patients with Osteoarthritis: Development and Nationwide Implementation of an Evidence‐Based Supported Osteoarthritis Self‐Management Programme. Musculoskeletal Care. 2015;13(2):67-75. doi:10.1002/msc.1085. 25345913

[7] GLA:D Canada – Managing Hip and Knee Osteoarthritis. Accessed June 25, 2024. https://gladcanada.ca/

[8] Von Elm E Altman DG Egger M Pocock SJ Gøtzsche PC Vandenbroucke JP STROBE Initiative . The Strengthening the Reporting of Observational Studies in Epidemiology (STROBE) statement: guidelines for reporting observational studies. J Clin Epidemiol. 2008;61(4):344-349. doi:10.1016/j.jclinepi.2007.11.008. 18313558

[9] Saskatchewan Centre for Patient-Oriented Research . Saskatchewan Centre for Patient-Oriented Research; 2025. https://www.scpor.ca. Accessed 21 June 2025.

[10] Osteoarthritis Program . Craven SPORT services, Saskatoon. Craven SPORT Services. https://cravensportservices.ca/physiotherapy/osteoarthritis-program-saskatoon/. Accessed 25 June 2024.

[11] Harris PA Taylor R Thielke R Payne J Gonzalez N Conde JG . Research electronic data capture (REDCap)—A metadata-driven methodology and workflow process for providing translational research informatics support. J Biomed Inform. 2009;42(2):377-381. doi:10.1016/j.jbi.2008.08.010. 18929686 PMC2700030

[12] Harris PA Taylor R Minor BL , et al. The REDCap consortium: Building an international community of software platform partners. J Biomed Inform. 2019;95:103208. doi:10.1016/j.jbi.2019.103208. 31078660 PMC7254481

[13] du Plessis V Beshiri R Bollman R . Definitions of ‘“rural.”; 2002. Published online.

[14] Weir CB Jan A . BMI Classification Percentile And Cut Off Points. In: StatPearls. StatPearls Publishing; 2025. https://www.ncbi.nlm.nih.gov/books/NBK541070/. Accessed 10 May 2025.

[15] NVivo Leading Qualitative Data Analysis Software (QDAS) by Lumivero. Lumivero. Accessed August 30, 2024. https://lumivero.com/products/nvivo/

[16] Young JJ Perruccio AV Veillette CJH McGlasson RA Zywiel MG . The GLA:D® Canada program for knee and hip osteoarthritis: A comprehensive profile of program participants from 2017 to 2022. In: Özden F , (ed). 2023;18(8):e0289645. doi:10.1371/journal.pone.0289645. PMC10399832 37535587

[17] Better Knee, Better Me, ^TM^: study team Bennell KL Keating C , et al. Better Knee, Better Me^TM^: effectiveness of two scalable health care interventions supporting self-management for knee osteoarthritis – protocol for a randomized controlled trial. BMC Musculoskelet Disord. 2020;21(1). doi:10.1186/s12891-020-3166-z. PMC7068989 32164604

[18] Hurley MV Walsh NE Mitchell H Nicholas J Patel A . Long‐term outcomes and costs of an integrated rehabilitation program for chronic knee pain: A pragmatic, cluster randomized, controlled trial. Arthritis Care Res. 2012;64(2):238-247. doi:10.1002/acr.20642. 21954131

[19] Liu X Seidel JE McDonald T , et al. Rural–Urban Disparities in Realized Spatial Access to General Practitioners, Orthopedic Surgeons, and Physiotherapists among People with Osteoarthritis in Alberta, Canada. Int J Environ Res Public Health. 2022;19(13):7706. doi:10.3390/ijerph19137706. 35805363 PMC9266058

[20] Osteoarthritis Self-Management - Exercise, Diet. Pain management. https://arthritis.ca/about-arthritis/arthritis-types-(a-z)/types/osteoarthritis/osteoarthritis-self-management

[21] Lee J Song J Hootman JM , et al. Obesity and other modifiable factors for physical inactivity measured by accelerometer in adults with knee osteoarthritis. Arthritis Care Res. 2013;65(1):53-61. doi:10.1002/acr.21754. PMC3449019 22674911

[22] White DK Jakiela J Bye T Aily J Voinier D . Stepping Forward: A Scoping Review of Physical Activity in Osteoarthritis. J Rheumatol. 2023;50(5):611-616. doi:10.3899/jrheum.220728. 36455947 PMC10159874

[23] Hinman RS Jones SE Nelligan RK , et al. Absence of Improvement With Exercise in Some Patients With Knee Osteoarthritis: A Qualitative Study of Responders and Nonresponders. Arthritis Care Res. 2023;75(9):1925-1938. doi:10.1002/acr.25085. 36594402

[24] Chou L Ellis L Papandony M , et al. Patients’ perceived needs of osteoarthritis health information: A systematic scoping review. In: Agarwal S , (ed), 2018;13:e0195489. doi:10.1371/journal.pone.0195489. PMC5901923 29659609

[25] Fernandes L Hagen KB Bijlsma JWJ , et al. EULAR recommendations for the non-pharmacological core management of hip and knee osteoarthritis. Ann Rheum Dis. 2013;72(7):1125-1135. doi:10.1136/annrheumdis-2012-202745. 23595142

[26] Brand C Cox S . Systems for implementing best practice for a chronic disease: management of osteoarthritis of the hip and knee. Intern Med J. 2006;36(3):170-179. doi:10.1111/j.1445-5994.2006.01018.x. 16503952

[27] Larson CO Nelson EC Gustafson D Batalden PB . The Relationship Between Meeting Patients’ Information Needs and their Satisfaction with Hospital Care and General Health Status Outcomes. Int J Qual Health Care. 1996;8(5):447-456. doi:10.1093/intqhc/8.5.447. 9117198

[28] Carmona-Terés V Moix-Queraltó J Pujol-Ribera E , et al. Understanding knee osteoarthritis from the patients’ perspective: a qualitative study. BMC Musculoskelet Disord. 2017;18(1):225. doi:10.1186/s12891-017-1584-3. 28558738 PMC5450398

[29] Ackerman IN Livingston JA Osborne RH . Personal Perspectives on Enablers and Barriers to Accessing Care for Hip and Knee Osteoarthritis. Phys Ther. 2016;96(1):26-36. doi:10.2522/ptj.20140357. 26206218

[30] Dobson F Bennell KL French SD , et al. Barriers and Facilitators to Exercise Participation in People with Hip and/or Knee Osteoarthritis: Synthesis of the Literature Using Behavior Change Theory. Am J Phys Med Rehabil. 2016;95(5):372-389. doi:10.1097/PHM.0000000000000448. 26945211

[31] Bath B Jakubowski M Mazzei D , et al. Factors Associated with Reduced Perceived Access to Physiotherapy Services among People with Low Back Disorders. Physiother Can. 2016;68(3):260-266. doi:10.3138/ptc.2015-50. 27909375 PMC5125465

[32] Ledingham A Cohn ES Baker KR Keysor JJ . Exercise adherence: beliefs of adults with knee osteoarthritis over 2 years. Physiother Theory Pract. 2020;36(12):1363-1378. doi:10.1080/09593985.2019.1566943. 30652930

[33] Picorelli AMA Pereira LSM Pereira DS Felício D Sherrington C . Adherence to exercise programs for older people is influenced by program characteristics and personal factors: a systematic review. J Physiother. 2014;60(3):151-156. doi:10.1016/j.jphys.2014.06.012. 25092418

[34] Dupuis F Perreault K Hébert LJ , et al. Group Physical Therapy Programs for Military Members With Musculoskeletal Disorders: A Pragmatic Randomized Controlled Trial. J Orthop Sports Phys Ther. 2024;54(6):417-426. doi:10.2519/jospt.2024.12342. 38530230

[35] Dupuis F Déry J Lucas De Oliveira FC , et al. Strategies to reduce waiting times in outpatient rehabilitation services for adults with physical disabilities: A systematic literature review. J Health Serv Res Policy. 2022;27(2):157-167. doi:10.1177/13558196211065707. 35156442

[36] Eichler S Rabe S Salzwedel A , et al. Effectiveness of an interactive telerehabilitation system with home-based exercise training in patients after total hip or knee replacement: study protocol for a multicenter, superiority, no-blinded randomized controlled trial. ReMove-It study group. Trials. 2017;18(1):438. doi:10.1186/s13063-017-2173-3. 28934966 PMC5608184

[37] Baruch Y Holtom BC . Survey response rate levels and trends in organizational research. Hum Relat. 2008;61(8):1139-1160. doi:10.1177/0018726708094863.

[38] Asch DA Jedrziewski MK Christakis NA . Response rates to mail surveys published in medical journals. J Clin Epidemiol. 1997;50(10):1129-1136. doi:10.1016/S0895-4356(97)00126-1. 9368521

[39] Fincham JE . Response Rates and Responsiveness for Surveys, Standards, and the Journal. Am J Pharm Educ. 2008;72(2):43. doi:10.5688/aj720243. 18483608 PMC2384218

[40] Smith MG Witte M Rocha S Basner M . Effectiveness of incentives and follow-up on increasing survey response rates and participation in field studies. BMC Med Res Methodol. 2019;19(1):230. doi:10.1186/s12874-019-0868-8. 31805869 PMC6896692

